# Factors associated with non-utilization of postnatal care among newborns in the first 2 days after birth in Pakistan: a nationwide cross-sectional study

**DOI:** 10.1080/16549716.2021.1973714

**Published:** 2021-09-17

**Authors:** Amir Saira, Leigh A. Wilson, Kingsley O Ezeh, David Lim, Uchechukwu L Osuagwu, Kingsley E Agho

**Affiliations:** a School of Health Sciences, Western Sydney University, Penrith, NSW, Australia; b Community Medicine department, Allama Iqbal Medical College, Lahore, Pakistan; c Faculty of Medicine and Health, School of Health Science, University of Sydney, Camperdown, NSW, Australia; d Translational Health Research Institute (THIR), School of Medicine, Western Sydney University, Campbelltown, NSW, Australia; e African Vision Research Institute, Discipline of Optometry, University of KwaZulu-Natal, Durban, South Africa

**Keywords:** Postnatal care, newborn, non-utilization, Pakistan, attributable risk

## Abstract

**Background:**

Recent data indicated that approximately four in every ten newborns in Pakistan do not receive postnatal care (PNC) services in the first 48 hours after delivery.

**Objectives:**

This study aimed to identify factors associated with the non-utilization of PNC for newborns in Pakistan using the 2017–18 Pakistan Demographic and Health Survey (PDHS).

**Methods:**

This was a cross-sectional analytical study utilizing data from 3887 live-born newborns recorded in the 2017–18 PDHS. Non-utilization of PNC was assessed against a set of independent factors using multilevel logistic regression analysis, and the population attributable risk estimates of factors associated with non-utilization of PNC were also calculated.

**Results:**

There were 1443 newborns (37%) in Pakistan whose mothers did not utilize PNC check-ups in the first 2 days after delivery. The non-utilization of PNC was largely attributable to newborns delivered at non-health facilities 53% (47% to 59%) and those born to uneducated women 27% (13% to 38%). Adjusted analyses indicated that newborns with higher birth order and with a birth interval of more than 2 years, women who perceived their baby to be small at birth, women with no formal education and those living in regional areas of Khyber Pakhtunkhwa and Federally Administered Tribal Areas were significantly associated with non-utilization of PNC services.

**Conclusions:**

Tailored health messages by community health workers, including door-to-door visits on utilizing health facilities through pregnancy to the postnatal periods, are needed and should target places of low socioeconomic status, including educationally disadvantaged women from regional areas of Pakistan.

## Background

Postnatal care was identified by the World Health Organization (WHO) as one of the key indicators in reducing childhood mortality and improving maternal health and is aligned with the United Nations Sustainable Development Goals 3 of good health and wellbeing [1]. The postnatal period (i.e. healthcare services received in the first 42 days after birth) is crucial for newborns survival as approximately 2.5 million newborns globally died in the first 28 days of life in 2018 and of these postnatal newborn deaths, close to 75% happened in the first 7 days after birth [2]. The majority of these postnatal newborn deaths occur in low- and middle-income countries (LMICs), including Pakistan, and are preventable or treatable if newborns receive timely and appropriate postnatal care (PNC) services recommended by the WHO [3], such as those linked to prematurity which accounts for almost 28% of newborn deaths [4].

The protective effect of PNC on newborn deaths has been recognized and documented in the public health literature. In response to the PNC benefits to the mother and infant, in the past decade and a half, the Pakistan Government has adopted and implemented a range of interventions such as the national Maternal, Neonatal, and Child Health (MNCH) initiative whose core aims include providing emergency obstetric services and community midwives and promoting institutional deliveries and skilled birth attendance [5]. Monitoring an infant’s growth, improved hygiene, and counselling for vaccination of mothers and infants were also part of these targeted interventions carried out by trained Lady Health Workers (LHWs) [5]. The LHWs are part of the healthcare workforce in Pakistan trained and deployed to provide primary health-care services through home visits, especially in rural and remote communities [5].

Despite all of these initiatives, a recent report from the Pakistan Demographic and Health Survey (PDHS) indicated that over the last decade, the prevalence of newborns that received PNC within 2 days after birth increased by approximately 62%, from 39.4% in 2006/7 to 63.9% in 2017/18 [5,6]. The current national PNC coverage can be interpreted as approximately four in every 10 newborns did not receive PNC within 2 days of delivery. This may be one of the contributing factors why Pakistan has the highest neonatal mortality rate (42 deaths per 1000 live births) in the Southern Asia region [5]. These statistics suggest the need for scaling up effective public health strategies and interventions directed at a national PNC coverage in Pakistan [5].

Studies on the non-utilization of PNC services have been conducted in many LMICs, including Nigeria [7,8], Indonesia [9], and Timor-Leste [10]. However, in Pakistan, the literature is very limited, and few of those past studies were either community-based or population-based studies that focused birth or populationon low utilization or utilization of PNC services [11–14]. Limitations are that prior to this study, there was no published non-utilization of PNC risk estimates for newborns within 2 days  of birth or population attributable risk (PAR) proportions adjusted for independent factors related to non-utilization of PNC services in Pakistan. In this study, we asked the question, what is the driver for the non-utilization of the PNC services in Pakistan? Therefore, the aim of this study was to examine the possible factors associated with non-utilization of PNC services among newborns within 2 days after birth using data from live births in the 2 years prior to the 2017–18 PDHS survey interview date. Adjusted PAR estimates were also obtained to measure the total magnitude of each of the significant independent risk factors related to non-utilization of PNC among newborns in Pakistan.

## Methods

Data regarding factors related to PNC services were extracted from a national household survey recorded in the 2017–18 PDHS [5]. The survey is carried out in Pakistan approximately every 5 years since 2006–7 by the National Institute of Population Studies (NIPS) in collaboration with Measure DHS ICF International (Calverton, MD, USA). The statistical methodology used in obtaining demographic and health characteristics has been described elsewhere [5]. Data concerning PNC services for the most recent births in the 2 years preceding the 2017–18 PDHS survey were reported to minimize women’s differential recall of events, as deliveries occurred at different periods in time prior to the survey date.

Data on PNC services usage were gathered from 12,364 eligible women (aged 15–49 years old) who were successfully interviewed during the survey. Sixty-two percent of these women had a total of 3,887 most recent live births in the 2 years prior to the survey data and were used for the current study analyses. Data collected from Azad Jammu and Kashmir, and Gilgit-Baltistan regions were not included in the study because those data were not incorporated in the total national estimates reported by the PDHS 2017–18 [5].

### Study outcome variable

The dependent variable for this study was the non-utilization of PNC services. This was partitioned into two mutually exclusive parts, such that non-utilization of PNC was considered as a ‘case’ (1 = if newborns had no postnatal check-ups in the first 2 days after delivery) or a ‘non-case (0 = if newborns had check-ups in the first 2 days after delivery).

### Potential independent variables

**Figure 1.**
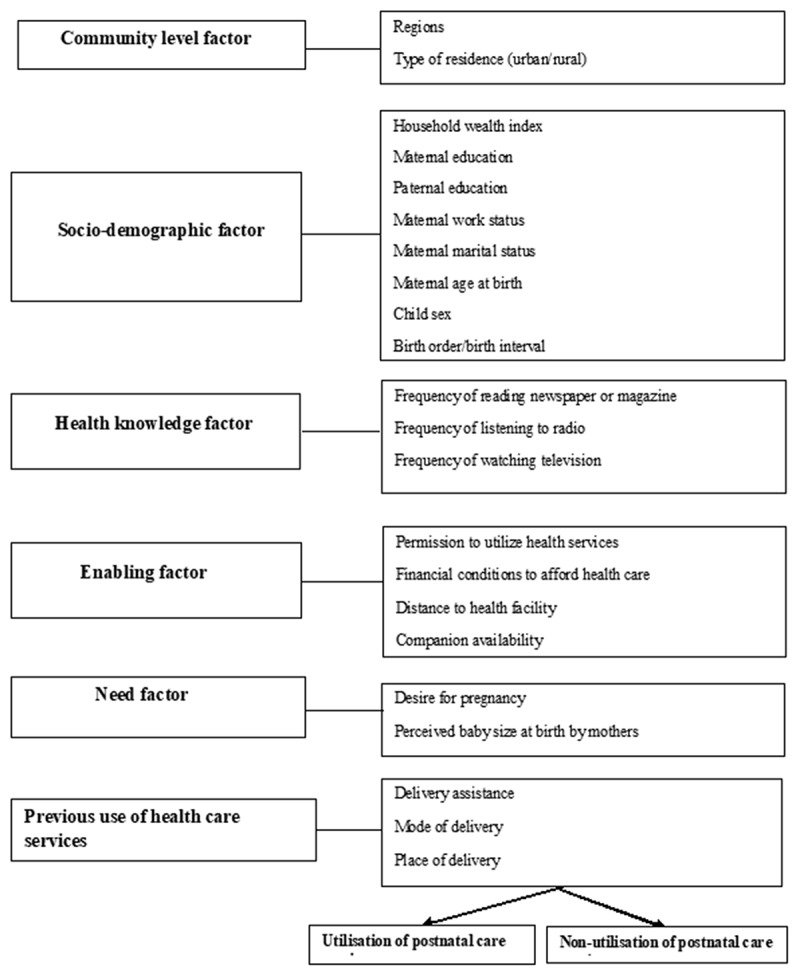
The study theoretical framework was adapted from Andersen’s behavioral model

Potential independent variables considered were based on similar past literature on non-utilization of PNC services in LMICs [7–9] and Andersen’s [15] behavioral theoretical framework of maternal health services. All the potential coexisting factors (Figure 1) were based on data availability in the 2017–18 PDHS, and these factors were categorized into six discrete groups: community-level factors, socio-demographic factors, health knowledge factors, enabling factors, need factors, and previous use of health services. The community-related confounders were residence type and region categorized into six groups (Punjab, Sindh, Balochistan, Islamabad capital territory (ICT), Khyber Pakhtunkhwa, and Federally Administered Tribal Areas (FATA). The residence type classified as (rural or urban) was included in the analysis because findings from earlier studies showed that living in rural areas was significantly associated with non-utilization of PNC [7,16]. Adherence to religious beliefs and cultural practices (e.g. keeping a newborn indoors for a specified period, particularly newborns delivered at non-health facilities) is more common in rural areas than in urban settings. Seclusion, part of cultural practices during the postnatal period, has been reported in past literature [17,18].

The possible independent socio-demographic level factors assessed consist of mother’s level of education, household wealth index, father’s level of education, mother’s age at birth, marital status, child sex, birth order/birth interval, and mother’s working status. A mother’s educational attainment has been previously reported to be strongly correlated with non-utilization of PNC [7,19,20]. It has been suggested that uneducated mothers may not be able to understand the full benefits of maternal health services and are less likely to appropriately utilize them than their educated counterparts. Mothers’ educational level was classified into three categories (no education, primary and secondary or higher education). Educated mothers, in turn, were more likely to obtain paid employment, which may likely increase access to modern health facilities. Hence, the inclusion of working status in the study analysis. Working status was divided into two groups (working or not working). We also adjusted for the mother’s age at the time of birth because it has been earlier opined that young maternal age (<20 years), inexperience in child-rearing and poor use of maternal health services have been attributed to morbidity and mortality of newborns [21]. It is possible that the infrequent use of maternal health services may vary as mothers get older. Maternal age was grouped into four classes (age <20, 20–29, 30–39 and 40–49 years).

Findings from recent studies in Nepal [22] and Bangladesh [23] indicated that there is a strong relationship between PNC services usage and rich household, implying that households of low socioeconomic status were less likely to patronize PNC services. Accordingly, the household wealth index was incorporated, and it was grouped into three classes (rich, middle and poor). The household wealth index in the 2017–2018 PDHS was constructed using a weighted factor score based on household facilities and assets available to the respondents at the time of the survey [5]. Facilities and assets included were the type of floor material used in rooms, ownership of agricultural land, electricity, television, radio, refrigerator, telephone, car, bicycle, motorcycle and canoe, and a livestock farm or a bank account.

Health knowledge factors (listening to the radio, watching television and reading newspapers or magazines) were also included in the study because previous studies [7,9,24] have shown that mothers who had inadequate or no access to mass media had a significantly increased odds of non-utilization of PNC services. Information through mass media can enhance mother’s knowledge and improve their ability to seek timely health-care services [25].

In the 2017–2018 PDHS, women participants (15–49 years) were asked if the following enabling factors ‘(getting permission to seek medical advice, getting money for advice or treatment, distance to a health facility, not wanting to go alone)’ constituted hindrance in accessing health-care services for themselves. The prevalence report showed that 58%, 42%, 30% and 21% of women reported not wanting to go alone, distance to the health facility, getting money for treatment, and getting permission to seek medical advice, respectively, encumbered their ability to access healthcare services. As a result of these statistics, we considered enabling factors in the study analysis. Need factors such as perceived newborn’s size at birth by mothers and desire for pregnancy were also included because similar studies by Agho et al. [7] and Titaley et al. [9] opined that smaller sized newborns had a significantly increased likelihood of non-utilization of PNC services. Perceived newborn’s size was classified into three groups (small, average and large). It was utilized as a proxy for the actual newborn’s weight at birth due to over 50% were not weighed at birth. This proxy approach was not unreasonable due to an earlier study that has indicated that there exists a close relationship between mean birth weight and perceived newborn size by the mother [26]. Mode of delivery, place of delivery and delivery assistance were grouped as previous use of health-care services. There was inconsistency in the relationship between place of delivery and non-utilization of PNC services; for example, Somefun and Ibisomi, in their study, revealed that newborns delivered in a non-health facility (or home facility) were more likely to use PNC services [27]. Whilst other studies suggested that newborns delivered in non-health facilities were less likely to use PNC services [9,16,28]. As a result of this inconsistency, we included the place of delivery in the current study.

## Statistical analysis

A frequency tabulation of non-utilization of PNC services for all possible coexisting variables included in this study was initially assessed. The estimation of the crude odd ratios (ORs) and adjusted ORs (aOR) that measures the strength of the study variables associated with non-utilization of PNC services was then investigated using the bivariable and multivariable logistics regression model.

The multivariable analysis was undertaken using a stage modelling approach similar to that described by Dibley et al. [26]. Modelling for each level factor (Figure 1) was done separately to examine their relationship to the study outcome. In all, five-stage modelling was conducted following an adopted conceptual framework (Figure 1).

In the first stage, community-level variables were entered into the baseline model, and a manually stepwise backward elimination process was undertaken to identify variables significantly associated with the study outcome at a 5% significance level were retained. In the second stage, socio-demographic level factor variables were investigated with the community variables that were significantly related to non-utilization of PNC, and those variables with p values <0.05 were retained as before. In the third, fourth and fifth stages, a similar process was used for enabling, need and previous use of health services level factors, respectively. Collinearity was assessed in our final model to minimize any statistical bias. STATA V.13.1 (STATA Corporation, College Station, Texas, USA) was employed for all the study analyses. The PDHS cluster sampling survey design and weights were adjusted for with ‘SVY’ STATA command.

Estimation of the PAR proportions and 95% confidence interval (CI) were calculated based on a similar approach used in previous studies [7,9,29]. The PAR estimate was used to measure the magnitude of risk attributed to each significant variable to the overall risk for non-utilization of PNC between 2012–13 and 2017–18.

## Results

Of the 3,887 newborns delivered within 2 years preceding the 2017–18 PDHS survey interview date, approximately 37% of the newborns (n = 1443) did not receive any PNC check-up in the first 2 days after delivery. Over three-quarters of babies delivered at non-health facilities (76.5%; 95% CI: 72%–80.5%) did not receive PNC services during the first 2 days of life, and approximately 70% of newborns who assisted by non-health professionals during birth (70.4%; 95% CI: 65.8%–74.7%) did not use PNC services. Fifty percent of newborns of non-educated mothers (95% CI: 45.8%–54.3%) did not utilize PNC services (Table 1).

**Table 1.** Distribution of characteristics, unadjusted and adjusted odd ratios (OR) for factors associated with non-utilisation of postnatal care services in Pakistan, 2017–18 Demographic and Health Survey (PDHS)

			Unadjusted‡	Adjusted‡
Variable	N	%*(95%CI)	OR (95%CI)	OR (95%CI)
*Community factor*				
**Residence type**				
Urban	1285	24.4(20.9–28.4)	Ref	
Rural	2602	43.4 (39.2–47.7)	2.37 (1.81–3.11)	
**Region**				
Punjab	2049	32.5 (27.2–38.3)	Ref	Ref
Sindh	903	27.3 (22.8–32.4)	0.78 (0.55–1.11)	0.64 (0.45–0.91)
Khyber Pakhtunkhwa	620	55.6 (49.3–61.7)	2.60 (1.82–3.71)	2.33 (1.65–3.30)
Balochistan	197	60.8 (53.5–67.6)	3.22 (2.18–4.76)	1.43 (0.92–2.20)
Islamabad (ICT)	31	21.5 (15.7–27.2)	0.57 (0.38–0.85)	0.82 (0.55–1.22)
FATA	87	68.3 (60.2–75.4)	4.46 (2.89–6.89)	3.36 (2.19–5.15)
*Socio-demographic factor*				
**Household wealth index**				
Rich	752	13.8 (10.6–17.9)	Ref	
Middle	1558	33.0(29.0–37.3)	3.07 (2.19–4.29)	
Lower	1578	52.3 (47.5–57.1)	6.83 (4.74–9.82)	
**Mother’s education**				
Secondary or higher	1436	21.0 (17.7–24.6)	Ref	Ref
Primary	613	36.4 (30.7–42.5)	2.16 (1.59–2.93)	1.49 (1.06–2.09)
No education	1839	50.0 (45.7–54.3)	3.77 (2.88–4.94)	1.72 (1.28–2.30)
**Mother’s working status**				
Working	486	35.0(28.7–42.0)	Ref	
Not-working	3401	37.4 (34.0–41.0)	1.11 (0.82–1.50)	
**Mother’s age**				
<20	355	46.0 (39.1–53.1)	Ref	
20–34	3118	35.2 (31.8–38.8)	0.82 (0.68–0.99)	
35–49	415	44.2 (37.0–51.6)	1.27 (0.92–1.75)	
**Marital status**				
Currently married	3851	37.3(34.0–40.6)	Ref	
Formerly/never married	36	23.9 (9.8–47.5)	0.52 (0.18–1.53)	
**Father’s education**				
Secondary/higher	2109	28.2 (24.8–31.8)	Ref	
Primary	630	42.1 (35.7–48.7)	1.85 (1.43–2.40)	
No education	1102	51.6 (47.1–56.1)	2.72 (2.15–3.43)	
**Birth rank and birth interval**				
2 or 3 child, interval >2	1245	32.3(28.1–36.7)	Ref	Ref
First child	927	31.8 (26.9–37.2)	0.98 (0.76–1.25)	1.37(1.01–1.85)
2 or 3 child, interval≤2	817	35.2 (30.7–39.9)	1.14 (0.90–1.44)	1.25 (0.94–1.67)
4 or more child, interval>2	595	51.1 (45.6–56.6)	2.19 (1.69–2.85)	1.62 (1.16–2.28)
4 or more child, interval≤2	302	51.4 (43.7–58.9)	2.22 (1.54–3.20)	1.41 (0.87–2.27)
**Child sex**				
Male	1955	35.9 (32.1–40.0)	Ref	
Female	1932	38.3(34.8–42.0)	1.11 (0.94–1.30)	
*Health knowledge factor*				
**Frequency of reading newspaper or magazine**				
At least once a week	186	14.3(8.83–22.3)	Ref	
Less than once a week	334	24.2(19.1–30.2)	1.92(1.07–3.44)	
Never	3365	39.7(36.3–43.2)	3.95 (2.23–6.98)	
**Frequency of listening to radio**				
At least once a week	147	28.5(20.7–37.8)	Ref	
Less than once a week	178	33.6(24.2–44.5)	1.27(0.72–2.25)	
Never	3563	37.7(34.3–41.2)	1.52 (0.98–2.37)	
**Frequency of watching TV**				
At least once a week	1819	26.8(23.2–30.8)	Ref	
Less than once a week	436	29.4(23.2–36.5)	1.14(0.81–1.60)	
Never	1632	50.7(46.4–55.0)	2.81 (2.19–3.60)	
*Enabling factor*				
**Seek permission to visit health services**				
Not a big problem	2975	33.8(30.1–37.6)	Ref	
Big problem	909	48.3 (43.4–53.2)	1.83 (1.45–2.32)	
**Getting money to pay for health services**				
Not a big problem	2637	31.7 (28.1–35.5)	Ref	
Big problem	1247	48.7(44.6–52.9)	2.05 (1.67–2.51)	
**Distance to health facility**				
Not a big problem	2111	30.8 (27.1–34.7)	Ref	
Big problem	1773	44.7 (40.3–49.3)	1.82 (1.44–2.31)	
**Want to be accompanied to health facility**				
Not a big problem	1370	32.5(28.1–37.2)	Ref	
Big problem	2515	39.7(35.9–43.7)	1.37 (1.08–1.74)	
*Need factor*				
**Wanted pregnancy at the time**				
Wanted then	3363	37.1(33.6–40.6)	Ref	
Wanted later	308	34.0 (27.4–41.4)	0.88 (0.63–1.23)	
Unwanted	216	42.6 (33.7–52.0)	1.26 (0.87–1.83)	
**Perceived baby size by their mother**				
Large	239	31.1(24.5–38.7)	Ref	Ref
Average	2780	35.3(32.0–38.8)	1.21(0.86–1.70)	1.19(0.79–1.79)
Small	857	44.0(39.0–49.2)	1.74 (1.24–2.43)	1.65 (1.10–2.47)
*Previous use of health services factor*				
**Delivery assistance**				
Health professional⸇	2602	21.1 (18.6–23.8)	Ref	
Non-health professional±	1243	70.4 (65.8–74.7)	8.21 (6.33–10.7)	
**Mode of delivery**				
Non-caesarean	2884	47.6 (44.1–51.2)	Ref	
Caesarean section§	68	6.8 (4.8–9.5)	0.08 (0.06–0.12)	
**Place of delivery**				
Healthcare facility	2761	21.3 (18.9–23.9)	Ref	Ref
Non-healthcare facility¥	1114	76.5 (72.0–80.5)	12.1 (9.25–15.7)	10.4 (7.78–13.9)

N, weighted total number of babies eligible for postnatal care services across each of the categories between 2012–13 and 2017–18 as reported by mothers interviewed during the survey; OR, odd ratio; CI, confidence interval; Ref, reference category; *, weighted prevalence of babies who did not receive any postnatal check during the first 2 days after birth; FATA, Federally Administered Tribal *Areas; TV, television; ICT, Islamabad capital territory;* §, Caesarean section is a combination of elective and emergency procedures; ‡,missing values were excluded from the mode;. ¥, refers to home facility; ±, Dai or traditional birth attendant; ⸇, doctor, nurse, midwife, lady health visitor, or community midwife.

In the final model of the multivariable analyses (Supplementary file: Table S1) it was indicated that newborns of mothers who resided in FATA (aOR = 3.36; 95% CI: 2.19–3.15) or Khyber Pakhtunkhwa (aOR = 2.33; 95% CI: 1.65–3.30) had a significantly greater odds of not utilizing PNC services compared with those living in Punjab areas. Collinearity investigation revealed that when the region was substituted by residence type in the final model, there was a significantly increased likelihood of non-utilization of PNC services for newborns of residing in rural areas (aOR = 1.63, 95% CI: 1.18–2.23). Newborns perceived by their mothers as small had a higher odds of not utilizing PNC services (aOR = 1.65; 95% CI: 1.10–2.47) after birth, as were newborns delivered at non-health facilities (aOR = 10.4; 95% CI: 7.78–13.9). A similar finding was also noted when we replaced place of birth with delivery assistance in the final model; newborns delivered by non-health professionals were less likely to utilize PNC services (aOR = 3.14, 95% CI: 2.41–4.10)

Other factors that were significantly related to the non-utilization of PNC services during the study period were newborns born to mothers who had primary or no formal education (aOR = 1.72, 95% CI: 1.28–2.30), and newborns of a fourth or higher-order with a birth interval more than 2 years (Table 1). A strong statistically significant relationship was also noted when mothers' educational levels were replaced one at a time by household wealth index and paternal educational level in the final model, newborns in poor households (OR = 2.46, 95% CI: 1.60–3.79) and newborns from uneducated fathers (OR = 1.44, 95% CI: 1.07 – .94) had increased odds of non-utilization of PNC services.

Of the total estimated risk for non-utilization of PNC services between 2012–13 and 2017–18 in Pakistan, 53% was attributable to newborns delivered at non-health facilities (PAR = 0.53, 95% CI: 0.47–0.59). During the same period, 27% of non-utilization of PNC in the first 2 days following delivery was attributed to newborns whose mothers had no formal education (PAR = 0.27, 95% CI: 0.13–0.38) (Table 2)

**Table 2.** Population attributable risk (PAR) for adjusted significant factors

Variable	%~	Adjusted‡† OR	PAR**	PAR (95%CI)
**Region**				
Punjab	0.46	1.00		
Sindh	0.17	0.64	–	
Khyber Pakhtunkhwa	0.24	2.33	0.14	0.14 (0.0–70.20)
Balochistan	0.08	1.43	–	–
Islamabad (ICT)	0.05	0.82	0.03	0.03 (0.0–20.05)
FATA	0.04	3.36	–	
**Mother’s education**				
Secondary or higher	0.21	1.00		
Primary	0.15	1.49	0.05	0.05 (0.01–0.10)
No education	0.64	1.72	0.27	0.27 (0.13–0.38)
**Birth rank and birth interval**				
2 or 3 child, interval >2	0.28	1.00		
First child	0.20	1.37	0.05	0.05 (0.00–0.11)
2 or 3 child, interval≤2	0.20	1.25	–	–
4 or more child, interval>2	0.21	1.62	0.08	0.08 (0.02–0.13)
4 or more child, interval≤2	0.11	1.41	–	–
**Perceived baby size by their mother**				
Large		1.00		
Average	0.69	1.19	–	
Small	0.26	1.65	0.10	0.10 (0.22–0.29)
**Place of delivery**				
Healthcare facility⸇	0.41	1.00		
Non-healthcare facility¥	0.59	10.4	0.53	0.53 (0.47–0.59)

~, weighted proportion of babies who did not receive postnatal check-up between 2012–13 and 2017–18 PDHS; OR, odd ratio; CI, confidence interval; FATA, Federally Administered Tribal Areas; ICT, Islamabad capital territory; ‡, missing values were excluded from the model; –, PAR was not obtained because factors were not significantly associated with non – utilization of postnatal care; **, PAR was calculated using similar formula described by previous studies [11,13,21], that is, PAR = ~ × ((adjusted OR−1)/adjusted OR); †, the adjusted model included place of residence; region; household wealth index; mother’s education, working status, age, marital status, father’s education, birth order and birth interval, child sex, frequency of (reading newspaper/magazine, listening to radio, watching television), permission to visit health services, money to pay for health services, distance to health facility, want to be accompanied to health facility, desire for pregnancy; place of birth; delivery assistance; mode of delivery and child’s body size at birth; ¥, refers to home facility; ⸇, doctor, nurse, midwife, lady health visitor, or community midwife.

## Discussion

In Pakistan, the overall national prevalence of non-utilization of PNC services moderately declined from approximately 57% in 2012–13 to approximately 37% in 2017–18 [5,30], and it remains uneven across regions. The variation in the prevalence of non-utilization of PNC among regions in Pakistan may be due to geographical location, socio-demographics and health inequalities, as well as economic issues among different populations. This study found that non-utilization of PNC services in Pakistan during the study period was significantly associated with regions (Khyber Pakhtunkhwa and the FATA), maternal education (no formal education or primary), mothers’ perceived baby size at birth (small), birth order/interval (four or more child, interval >2 years), and delivery place (non-health facility). Therefore, further improvement is urgently needed in order to reduce the current neonatal mortality rate (42 deaths per 1000 live births) [5] to the United Nations Sustainable Development Goal target of 12 deaths per 1000 births by the year 2030 [31].

This study indicated that newborns born to mothers residing in Khyber Pakhtunkhwa and the FATA regions were less likely to use PNC within the first 48 hours of delivery. It has been previously suggested that regional-specific variations in maternal health-care services (e.g. ANC and PNC) usage may be attributed to differences in health care skilled health-care providers, education, access and distance to health-care facilities, as well as differences in regional economies [32,33]. However, a plausible reason for the higher odds of non-utilization of PNC in Khyber Pakhtunkhwa and the FATA regions may be attributed to their difficult geographical terrains (e.g. mountains, valleys, hills), resulting in inadequate access to maternal health-care services. This finding was supported by a recent study conducted in a mountainous area of Nepal, which suggested that underutilization of maternal health-care services may be linked to lack of transport and poor quality or limited road infrastructure in this area [34]. At the regional level in Pakistan, further research examining the characteristics of non-utilization of PNC may be needed to inform the more robust and effective distribution of maternal and child health (MCH) resources and policies to improve PNC usage within 48 hours of birth.

Findings from this study indicated that the risk of non-utilization of PNC services within 48 hours of birth was higher among newborns whose mothers had no formal education or only primary education (or limited education) compared with those with secondary or higher education. This finding is similar to previous outcomes suggested in Nigeria [7], Nepal [19] and India [20]. A plausible reason for this may be that uneducated mothers or mothers with a limited level of education are indecisive in making appropriate judgements on their health and utilization of maternal health-care services. Additionally, uneducated nursing mothers or mothers with limited education lack autonomy and do not integrate well with different economic and social settings, resulting in a low level of awareness and acceptance of new ideas concerning PNC usage. Evidence from previous studies [23,35] has shown that educated mothers are better informed about health risks and are more likely to seek and gain access to appropriate health-care services.

The increased likelihood of non-utilization of PNC services within 48 hours of birth was greater among newborn babies whose mothers perceived their size to be small-sized compared with those perceived as larger-sized. This finding is similar to cross-sectional studies conducted in Nigeria [7] and Indonesia [9], which suggested that smaller than average-sized newborns were less likely to receive PNC services. Perceived vulnerability (i.e. immaturity) of small-sized newborns could also have contributed to the mother’s inability to visit health facilities for PNC services. Therefore, promoting and supporting home-based PNC services could assist in providing crucial newborn care, especially for high-risk newborns. Furthermore, we noted that newborns of birth order (2 through 4 or higher) born with a birth interval (>2 years) were less likely to use PNC services. This finding contradicts an earlier study [7], which reported a statistically insignificant relationship between non-utilization of PNC services and infants of high birth rank. Nonetheless, the decrease utilization of PNC services among greater birth order babies has also been reported in Indonesia [9] and Peru [36, 37]. Inadequate time, as well as household economic limitations due to having many other infants, may be contributing reasons for the declined PNC usage among the higher birth order infants.

This study indicated that infants delivered in a non-healthcare facility had a decreased PNC utilization compared with those delivered in a health-care facility. Our outcome differs from a recent similar study [27] which suggested that infants delivered in a non-healthcare facility were significantly more likely to use PNC services. However, our finding is consistent with previous population-based studies in Nepal [16], Indonesia [9], and Zambia [28]. A plausible explanation for our finding may be the fact that women who delivered in a health-care facility were more exposed to maternal health education related to PNC services prior to and post-birth. Additionally, a combination of other possible factors, such as cultural, transportation difficulties, financial barriers and inadequate community providers for routine home visitation, could also contribute to a high level of non-utilization of PNC services in the first 48 hours post-birth.

A number of limitations were noted in the current study, which include (a) a causal relationship was not established between the study factors and non-utilization of PNC, as predictors of non-utilization of PNC were identified using cross-sectional data; (b) even though our study was restricted to birth within 2 years prior to the survey data, it is still possible that recall and measurement errors may have affected our current findings; (c) unmeasured potential coexisting factors, such as cultural beliefs, and family dynamics may have also impacted upon the study results. In Nepal, for example, traditional cultural practices have been reported to prevent mothers and newborns from being touched by anyone or leaving the house before the 12^th^ day after birth [37] and have been related to the non-utilization of PNC [32].

Despite the outlined limitations, there are important strengths of this study, including (1) the findings were based on nationally representative household data, and hence results can be generalized across all the regions in Pakistan; (2) a large sample size of newborn babies reported by mothers was used, resulting in the minimal potential effect of selection bias and unlikely to impact the findings.

## Conclusions

Our findings suggest that delivery at non-healthcare facilities, maternal education (no formal education), small baby size at birth, region (Khyber Pakhtunkhwa and the FATA) and child spacing were significantly associated with newborns not receiving PNC services in the first 2 days after birth in Pakistan. Based on the estimated PAR% it is recommended that effective public health interventions, especially strategic plans that discourage mothers from patronizing non-healthcare facilities for pregnancy and birth health-care services are needed. Also, focused support is needed for educationally disadvantaged mothers from rural and remote communities, particularly those living in areas with low PNC usage. Additionally, health promotion programs to create adequate community awareness of the importance of providing PNC services to small-sized newborns is required, and this may help in scaling down neonatal deaths in Pakistan.

## Supplementary Material

Supplemental Material

## Data sharing agreement

Data are available from the DHS programme (https://dhsprogram.com/).

## Paper context

The Pakistan 64% post-natal care (PNC) services for newborns is well below the WHO target of (≥80%) coverage by 2025, implying an urgent need to use standardized national data to guide effective uniform interventions across the regions. The increased likelihood of non-utilization of PNC was greater among newborns delivered at home and, from mothers with limited education. Interventional initiatives should educate women on the benefits of institutional delivery and focused support for low socioeconomic women.
