# Supplementary material for: Factors associated with non-utilization of postnatal care among newborns in the first 2 days after birth in Pakistan: a nationwide cross-sectional study
Source: Glob Health Action. 2021 Sep 17;14(1):1973714. doi: 10.1080/16549716.2021.1973714 (PMC8451633; doi:10.1080/16549716.2021.1973714)
Supplement: Supplemental Material [file ZGHA_A_1973714_SM7013.docx]

Supplementary table 1. All the multivariable analyses models of factors associated with postnatal care in Pakistan

| **Variable** | Model 0 | Model 1 | Model 2 | Model 3 | Model 4 | Model 5 | Model 6 |
| --- | --- | --- | --- | --- | --- | --- | --- |
| *Community factor* | OR (95%CI) | (95%CI) | (95%CI) | (95%CI) | (95%CI) | (95%CI) | (95%CI) |
| **Residence type** |  |  |  |  |  |  |  |
| Urban | Ref | Ref |  |  |  |  |  |
| Rural | 2.37(1.81-3.11) | 1.98(1.51-2.61)ⱽ |  |  |  |  |  |
| **Region** |  |  |  |  |  |  |  |
| Punjab | Ref | Ref | Ref | Ref | Ref | Ref | Ref |
| Sindh | 0.78(0.55-1.11) | 0.62 (0.43-1.01) | 0.69 (0.54-0.95) | 0.44 (0.30-0.65) | 0.51 (0.37-0.73) | 0.49 (0.33-0.71) | 0.64 (0.45-0.91) |
| Khyber Pakhtunkhwa | 2.60 (1.82-3.71) | 2.01 (1.77-2.63)ⱽ | 2.21 (1.91-3.13)ⱽ | 2.26 (1.73-2.87)ⱽ | 2.01 (1.49-2.21)ⱽ | 2.18 (1.35-2.50)ⱽ | 2.33 (1.65-3.30)ⱽ |
| Balochistan | 3.22 (2.18-4.76) | 2.22 (1.98-2.96)ⱽ | 1.17 (1.01-1.56)ⱽ | 1.09 (0.92-1.65) | 1.01 (0.87-1.72) | 1.08 (0.81-1.91) | 1.43 (0.92-2.20) |
| Islamabad (ICT) | 0.57 (0.38-0.85) | 0.99 (0.68-1.44) | 1.02 (0.81-1.37) | 1.03(0.79-1.27) | 0.74 (0.91-1.46) | 1.14 (0.79-1.65) | 0.82 (0.55-1.22) |
| FATA | 4.46 (2.89-6.89) | 3.46 (2.92-4.07)ⱽ | 2.56 (2.30-3.15)ⱽ | 2.45 (2.31-2.97)ⱽ | 2.23 (2.04-3.81)ⱽ | 2.79 (2.37-4.63)ⱽ | 3.36 (2.19-5.15)ⱽ |
| *Socio-demographic factor* |  |  |  |  |  |  |  |
| **Household wealth index** |  |  |  |  |  |  |  |
| Rich | Ref |  | Ref |  |  |  |  |
| Middle | 3.07 (2.19-4.29) |  | 1.27 (0.89-1.40) |  |  |  |  |
| Lower | 6.83 (4.74-9.82) |  | 2.27 (1.91-2.82)ⱽ |  |  |  |  |
| **Mother's education** |  |  |  |  |  |  |  |
| Secondary or higher | Ref |  | Ref | Ref | Ref | Ref | Ref |
| Primary | 2.16 (1.59-2.93) |  | 1.44 (1.01-2.04)ⱽ | 1.55 (1.09-2.20)ⱽ | 2.03 (1.43-2.88)ⱽ | 2.01 (1.41-2.85)ⱽ | 1.49 (1.06-2.09)ⱽ |
| No education | 3.77 (2.88-4.94) |  | 1.81 (1.32-2.48)ⱽ | 2.05 (1.51-2.80)ⱽ | 2.97 (2.20-4.02)ⱽ | 2.94 (2.21-3.90)ⱽ | 1.72 (1.28-2.30)ⱽ |
| **Mother's working status** |  |  |  |  |  |  |  |
| Working | Ref |  | Ref |  |  |  |  |
| Not-working | 1.11 (0.82-1.50) |  | 1.13 (0.78-1.64) |  |  |  |  |
| **Mother's age** |  |  |  |  |  |  |  |
| <20 | Ref |  | Ref |  |  |  |  |
| 20-34 | 0.82 (0.68-0.99) |  | 0.68 (0.47-1.07) |  |  |  |  |
| 35-49 | 1.27 (0.92-1.75) |  | 1.09 (0.87-1.67) |  |  |  |  |
| **Marital status** |  |  |  |  |  |  |  |
| Currently married | Ref |  | Ref |  |  |  |  |
| Formerly/never married | 0.52 (0.18-1.53) |  | 0.93 (0.79-1.31) |  |  |  |  |
| **Father's education** |  |  |  |  |  |  |  |
| Secondary/higher | Ref |  | Ref |  |  |  |  |
| Primary | 1.85 (1.43-2.40) |  | 1.24 (0.92-1.67) |  |  |  |  |
| No education | 2.72 (2.15-3.43) |  | 1.18 (0.92-1.51) |  |  |  |  |
| **Birth rank and birth interval** |  |  |  |  |  |  |  |
| 2 or 3 child, interval >2 | Ref |  | Ref | Ref | Ref |  | Ref |
| First child | 0.98 (0.76-1.25) |  | 0.79 (0.57-1.08) | 0.79 (0.57-1.08) | 1.18 (1.00-1.59) |  | 1.37(1.01-1.85)ⱽ |
| 2 or 3 child, interval<=2 | 1.14 (0.90-1.44) |  | 0.96 (0.71-1.30) | 0.96 (0.71-1.30) | 1.15 (0.88-1.52) |  | 1.25 (0.94-1.67) |
| 4 or more child, interval>2 | 2.19 (1.69-2.85) |  | 1.35 (1.19-1.68)ⱽ | 1.35 (1.19-1.68)ⱽ | 1.41 (1.26-1.91)ⱽ |  | 1.62 (1.16-2.28)ⱽ |
| 4 or more child, interval<=2 | 2.22 (1.54-3.20) |  | v1 78 (1.43-2.05) | 1.78 (1.43-2.05) | 1.35 (1.01-1.79)ⱽ |  | 1.41 (0.87-2.27) |
| **Child sex** |  |  |  |  |  |  |  |
| Male | Ref |  | Ref |  |  |  |  |
| Female | 1.11 (0.94-1.30) |  | 1.06 (0.87-1.28) |  |  |  |  |
| *Health knowledge factor* |  |  |  |  |  |  |  |
| **Frequency of reading newspaper or magazine** | |  |  |  |  |  |  |
| At least once a week | Ref |  |  | Ref |  |  |  |
| Less than once a week | 1.92(1.07-3.44) |  |  | 2.04(1.15-3.63) |  |  |  |
| Never | 3.95 (2.23-6.98) |  |  | 1.73 (1.00-3.01) |  |  |  |
| **Frequency of listening to radio** |  |  |  |  |  |  |  |
| At least once a week | Ref |  |  | Ref |  |  |  |
| Less than once a week | 1.27(0.72-2.25) |  |  | 0.98(0.58-2.28) |  |  |  |
| Never | 1.52 (0.98-2.37) |  |  | 1.16 (0.58-1.68) |  |  |  |
| **Frequency of watching TV** |  |  |  |  |  |  |  |
| At least once a week | Ref |  |  | Ref |  |  |  |
| Less than once a week | 1.14(0.81-1.60) |  |  | 1.03(0.71-1.50) |  |  |  |
| Never | 2.81 (2.19-3.60) |  |  | 1.13 (0.86-1.48) |  |  |  |
| *Enabling factor* |  |  |  |  |  |  |  |
| **Seek permission to visit health services** | |  |  |  |  |  |  |
| Not a big problem | Ref |  |  |  | Ref |  |  |
| Big problem | 1.83 (1.45-2.32) |  |  |  | 1.20 (0.87-1.65) |  |  |
| **Getting money to pay for health services** | |  |  |  |  |  |  |
| Not a big problem | Ref |  |  |  | Ref |  |  |
| Big problem | 2.05 (1.67-2.51) |  |  |  | 0.86 (0.64-1.15) |  |  |
| **Distance to health facility** |  |  |  |  |  |  |  |
| Not a big problem | Ref |  |  |  | Ref |  |  |
| Big problem | 1.82 (1.44-2.31) |  |  |  | 0.99 (0.74-1.33) |  |  |
| **Want to be accompanied to health facility** | |  |  |  |  |  |  |
| Not a big problem | Ref |  |  |  | Ref |  |  |
| Big problem | 1.37 (1.08-1.74) |  |  |  | 0.95 (0.70-1.27) |  |  |
| *Need factor* |  |  |  |  |  |  |  |
| **Wanted pregnancy at the time** |  |  |  |  |  |  |  |
| Wanted then | Ref |  |  |  |  | Ref |  |
| Wanted later | 0.88 (0.63-1.23) |  |  |  |  | 1.25 (0.83-1.84) |  |
| Unwanted | 1.26 (0.87-1.83) |  |  |  |  | 1.07 (0.61-1.75) |  |
| **Perceived baby size by their mother** | |  |  |  |  |  |  |
| Large | Ref |  |  |  |  | Ref | Ref |
| Average | 1.21(0.86-1.70) |  |  |  |  | 0.98 (0.67-1.43) | 1.19(0.79-1.79) |
| Small | 1.74 (1.24-2.43) |  |  |  |  | 1.35 (1.17-2.01)ⱽ | 1.65 (1.10-2.47)ⱽ |
| *Previous use of health services factor* | |  |  |  |  |  |  |
| **Delivery assistance** |  |  |  |  |  |  |  |
| Health professional⸇ | Ref |  |  |  |  |  | Ref |
| Non-health professional‡ | 8.21 (6.33-10.7) |  |  |  |  |  | 1.79 (0.98-2.87) |
| **Mode of delivery** |  |  |  |  |  |  |  |
| Non-caesarean | Ref |  |  |  |  |  | Ref |
| Caesarean section§ | 0.08 (0.06-0.12) |  |  |  |  |  | 0.31 (0.21-0.46) |
| **Place of delivery** |  |  |  |  |  |  |  |
| Healthcare facility | Ref |  |  |  |  |  | Ref |
| Non-healthcare facility¥ | 12.1 (9.25-15.7) |  |  |  |  |  | 10.4 (7.78-13.9)ⱽ |

Notes: ⱽ, significant variable (s) added to the next model; Model 0 – Unadjusted for all the independent variables; Model 1- Community level factors (residence type & region); Model 2 - significant variable(s) in Model 1 added to socio-demographic variables (household wealth status, maternal education, paternal education, mothers work status, child sex, mothers marital status, birth order/interval & mothers age); Model 3 – significant variable(s) in Model 2 added to health knowledge factor (frequency of reading newspaper/magazine, frequency of listening to radio & frequency of watching television); Model 4 – significant variables in Model 3 added to enabling factor (permission to seek medical advice, getting money to pay for health services, distance to health facility & want to be accompanied to health facility); Model 5- significant variables in Model 4 added to need factor (mother’s desire for pregnancy & mothers perceived baby size at birth); Model 6 – significant variables in Model 5 added to previous use of health service factor (delivery assistant, mode of delivery & place of delivery). aOR (95%CI): adjusted Odds ratio with corresponding 95% confidence interval, OR: unadjusted Odds ratio; ¥ refers to home facility; ‡, Dai or traditional birth attendant; Ref, reference category; ⸇, doctor, nurse, midwife, lady health visitor, or community midwife; §, Caesarean section is a combination of elective and emergency procedures.
